# Long noncoding RNAs (lncRNAs) in human lymphomas

**DOI:** 10.1016/j.gendis.2021.02.001

**Published:** 2021-02-12

**Authors:** Ali Gholami, Khosro Farhadi, Fatemeh Sayyadipour, Masoud Soleimani, Fakhredin Saba

**Affiliations:** a Clinical Research Development Center, Taleghani and Imam Ali Hospital, Kermanshah University of Medical Sciences, Kermanshah 671568-5420, Iran; b Department of Haematology, Tarbiat Modares University, Tehran 146899-5513, Iran; c Department of Medical Laboratory Science, School of Paramedical, Kermanshah University of Medical Sciences, Kermanshah 671568-5420, Iran

**Keywords:** Apoptosis, Long non-coding RNA, Lymphoma, Prognosis, Proliferation

## Abstract

Lymphomas are a diverse group of haematologic malignancies, which occur in infection-fighting cells of the lymphatic system. Long non-coding RNAs (lncRNAs) are non-coding RNAs, which have recently received significant attention as the main mediators of gene expression. In this review, we summarize the current knowledge on lncRNAs involved in lymphomas, their molecular functions, as well as their potential clinical value. Relevant literature was identified by a PubMed search of English language papers using the following terms: Lymphoma, LncRNA, leukemia, proliferation, apoptosis, and prognosis. LncRNAs are imperative for lymphoma carcinogenesis through affecting apoptosis, cell proliferation, invasion, and response to chemotherapy. The expression level of lncRNAs can affect chemotherapy-induced apoptosis. Taken together, lncRNA dysregulation in lymphoma cells is not only an epiphenomenon but also lncRNA transcription is critically related to the initiation and progression of lymphomas. Aberrant expression of lncRNAs can lead to the transformation of normal lymphocytes into lymphoma cells.

## Abbreviations

ABC: Activated B-cell
APC-1: Anaphase-promoting complex subunit −1
ALCL: Anaplastic large cell lymphoma
ANRIL: Antisense non-coding RNA in the INK4 locus
AURK: Aurora kinase
BAX: B-cell lymphoma 2-associated X
BCR: B-cell receptor
BL: Burkitt lymphoma
Bmi1: B-lymphoma Mo-MLV insertion region 1 homolog
CDK: Cyclin-dependent kinase
C/EBP β: CCAAT enhancer binding protein β
CERNA: Competitive endogenous RNA
CHL: Classical hodgkin lymphoma
DLBCL: Diffuse large B-cell lymphoma
ECM: Extracellular matrix
EMT: Epithelial-mesenchymal transitions
ERK: Extracellular-signal-regulated kinase
EZH2: enhancer of Zeste homolog 2
FAS: Fas cell surface death receptor
FL: Follicular lymphoma
FU: Fludarabine
GAS5: Growth arrest-specific 5
GCB: Germinal center B-cell
HOTAIR: HOX transcript antisense RNA
HULC: Hepatocellular carcinoma up-regulated long non-coding RNA
H3k27me3: Histone H3 at lysine 27
IAP: Inhibitor of apoptosis
IPI: International Prognostic Index
LC3: Light chain 3
LDH: Lactate dehydrogenase
LINK-A: Long intergenic non-coding RNA for kinase activation
LncRNA: Long non-coding RNA
LUNAR1: Leukemia-associated non-coding IGF1R activator RNA 1
MALAT1: Metastasis associated lung adenocarcinoma transcript 1
MAPK: Mitogen-activated protein kinase
MCL: Mantle cell lymphoma
MEF2C: Myocyte-specific enhancer factor 2C
MEG3: Maternally expressed gene 3
MEK: Mitogen-activated protein kinase/ERK kinase
MMP: Matrix metalloproteinase
mTOR: Mammalian target of rapamycin
Myc: Myelocytomatosis
NEAT2: Nuclear enriched abundant transcript 2
NF-κB: Nuclear factor kappa-light-chain-enhancer of activated B cells
NHL: Non-hodgkin lymphoma
NK: Natural killer
NLPHL: Nodular lymphocyte-predominant hodgkin lymphoma
NSCLC: Non-small cell lung cancer
PANDA: P21-associated ncRNA DNA damage-activated
PARP: Poly ADP-ribose polymerase
PCG: Polycomb group
PEG10: Paternally expressed 10
PRC: Polycomb repressive complex
PVT1: Plasmacytoma variant translocation1
RBM5: RNA binding motif protein5
ROR1: Receptor tyrosine kinase-like orphan receptor1
RS: Reed–Sternberg
SDCBP: Syndecan binding protein
SOX11: SRY-related HMG-Box 11
SUZ12: Suppressor of Zeste 12 homolog
T-LBL: T-lymphoblastic lymphoma
WHO: World health organization

## Introduction

**Figure 1:**
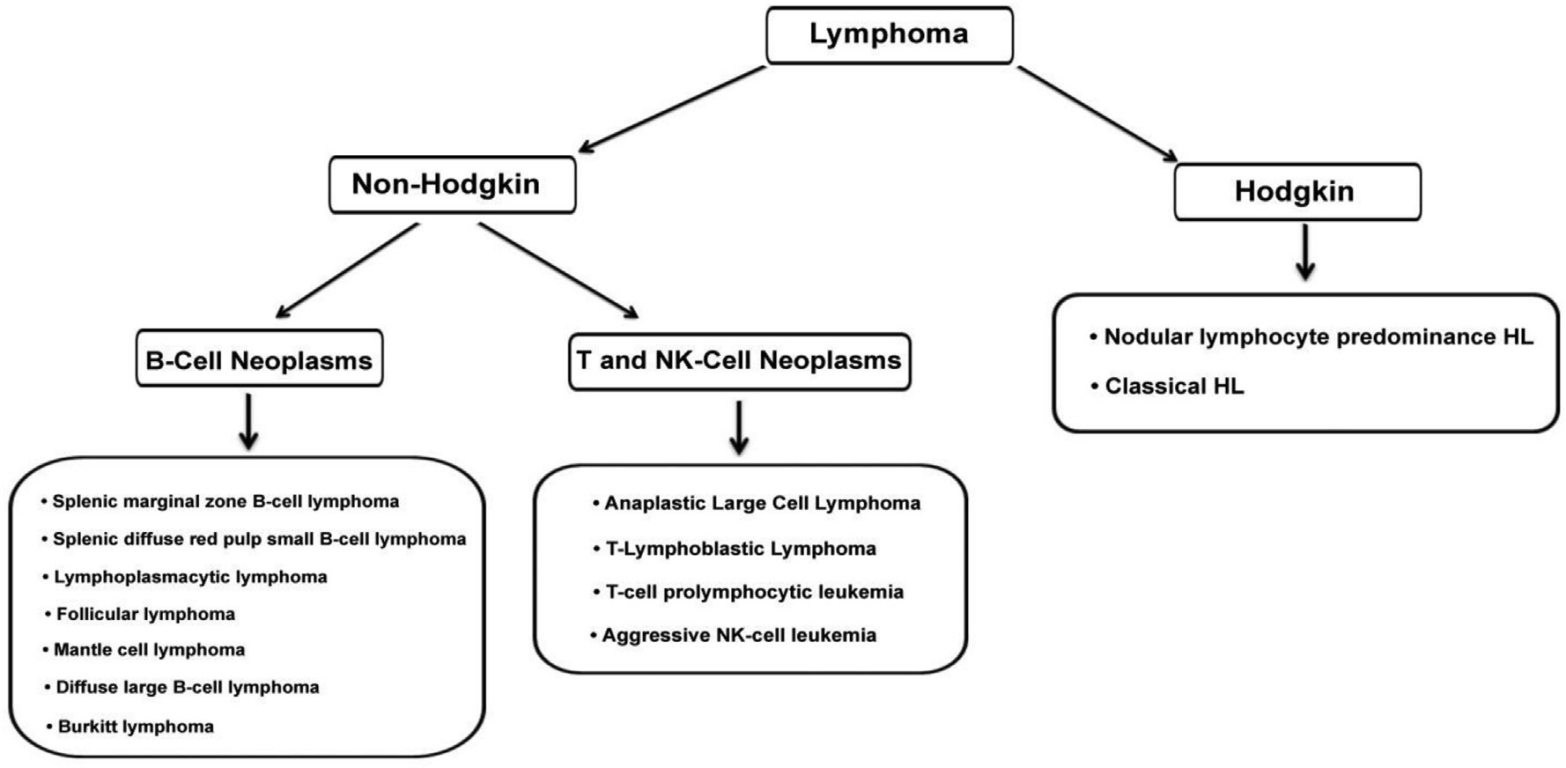
The lymphoma classification. Lymphomas are classified into two major groups: NHL and HL. The lymphoma subtypes are broadly based on the immunophenotypic features and morphologic and genetic characteristics in the context of clinical presentation. NHL, non-hodgkin lymphoma; NK, natural killer; HL, hodgkin lymphoma.

Lymphomas are hematologic malignancies, which occur in infection-fighting cells of the lymphatic system, called lymphocytes.^1^^,^^2^ Based on the presence of typical Reed–Sternberg (RS) cells, lymphomas are divided into two main groups; Hodgkin and non-Hodgkin lymphoma (HL and NHL), which differ in terms of their genetic mutations, clinical demonstrations, and treatment approaches (Fig. 1).^1^^,^^2^ HL is classified into nodular lymphocyte-predominant Hodgkin lymphoma (NLPHL) and classical Hodgkin lymphoma (cHL).^3^ CHL is the most predominant HL subtype, which accounts for nearly 95% of HL.^4^ The updated World Health Organization (WHO) classification of NHL is based on genetic, immunophenotypic, and clinical characteristics.^5^ Diffuse large B-cell lymphoma (DLBCL), follicular lymphoma (FL), Burkitt's lymphoma (BL), and mantle-cell lymphoma (MCL) are categorized into NHL groups (Fig. 1).^1^ Recently, much progress has been made regarding the research of long non-coding RNAs (lncRNAs) in lymphomas.^6^

**Figure 2:**
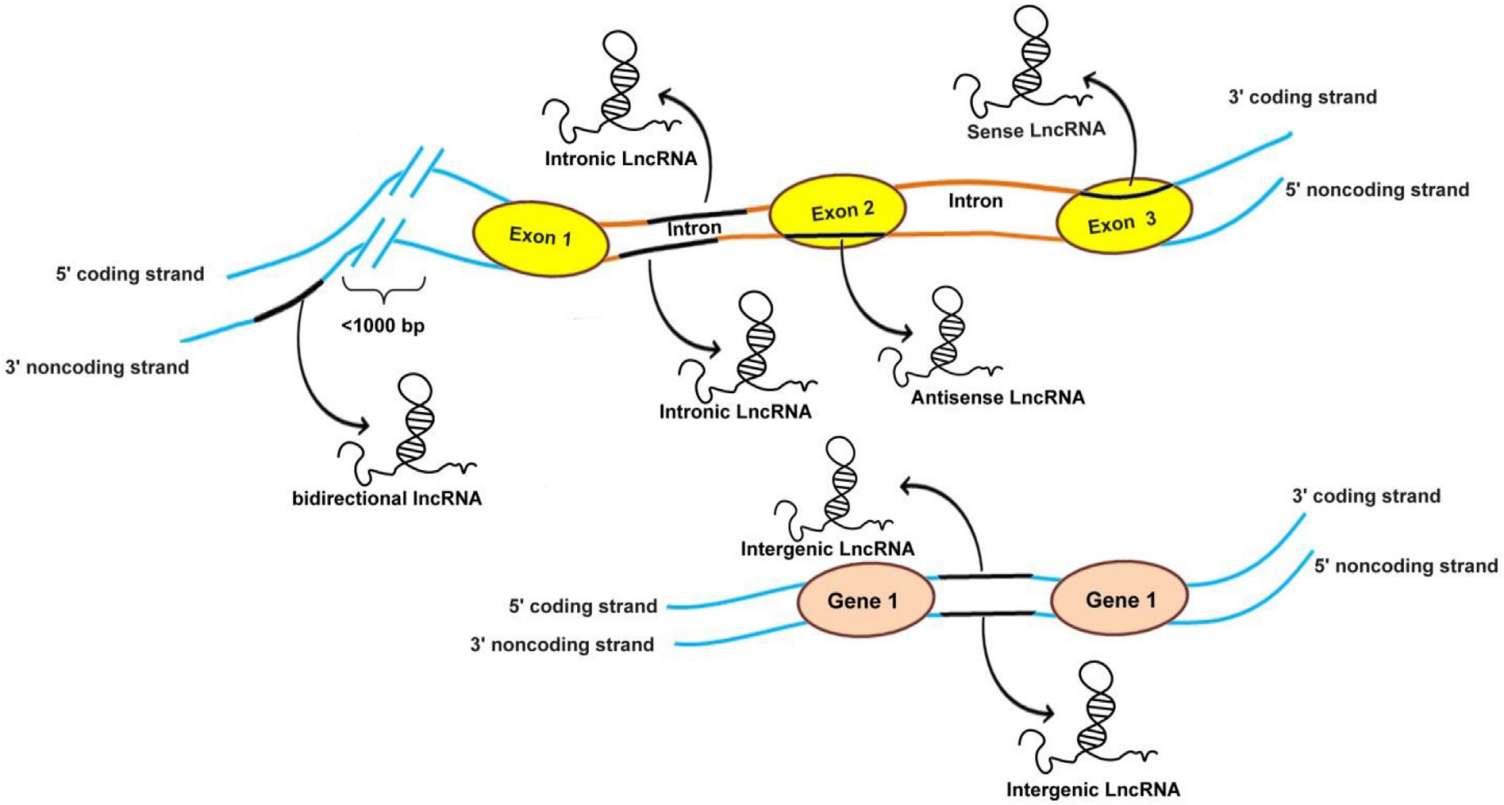
Overview of classification of lncRNAs. (a) Sense lncRNAs overlap with the sense strand of a protein-coding gene; (b) Antisense lncRNAs overlap with exons of a protein-coding gene on the opposite strand; (c) The expression of a bidirectional lncRNA on the opposite strand is initiated at <1000 base pairs away in close genomic proximity; (d) Intronic lncRNA is transcribed entirely within an intron of a protein-coding gene without overlapping exons; (e) Long intergenic non-coding RNAs (lincRNAs) are intergenically in both directions.

LncRNAs are approximately more than 200 nucleotides long and they are dynamically expressed in different cellular processes.^7^ LncRNAs may be transcribed from intergenic, genic, and enhancer regions.^8^^,^^9^ Some lncRNAs may have a promoter with the neighboring coding gene.^9^ LncRNAs similar to mRNAs are transcribed by RNA polymerase II, spliced, 5’ capped, and polyadenylated.^10^ Despite these similarities to mRNAs, lncRNAs indicate specific characteristics, which distinguish them from mRNAs.^10^ The major difference between mRNAs and lncRNAs is that lncRNAs are not translated into protein.^11^ LncRNAs have commonly lower expression, the fewer number of exons and show more specific expression in different tissues.^11^ Generally, lncRNAs are classified according to their orientation and position in the genome, including sense, antisense, intronic, intergenic, and bidirectional (Fig. 2).^9^ Sense and antisense lncRNAs overlap, partially or entirely, one or more exons of protein-coding genes.^9^ They are defined according to the nearest protein-coding genes positions.^9^ Sense lncRNAs are transcribed from the sense strand of protein-coding genes, while antisense lncRNAs are encoded from the antisense strand.^9^ Intronic lncRNAs are transcribed entirely from introns and do not overlap with any exon.^9^ Moreover, some lncRNA sequences are located within the protein-coding genes.^12^ These include two groups; the first group is bidirectional lncRNAs, which its transcription starts less than 1 kb from a protein-coding gene transcription start site, but on the opposite DNA strand.^12^ The other group includes long intergenic non-coding RNAs (lincRNAs), which are transcribed intergenically from both strands.^12^ LincRNAs can reach lengths of 1 Mbase.^12^

Although the discovery of the whole genome sequence has provided researchers the tools to know how genetic mutations lead to inappropriate cell functions, molecular mechanisms of lncRNAs are now being realized, and emerging methods are developing investigators' abilities to functionally annotate cancer-related lncRNAs. Recent studies have shown that lncRNAs can intervene in the regulation of cell proliferation, apoptosis, and maintenance of stemness during cancer development.^13^ Furthermore, recent reports have indicated that lncRNA may also engage in remodeling the tumor microenvironment, which predicts tumor behavior and disease prognosis.^13^

Multiple studies have demonstrated the expression changes of lncRNAs in various stages of lymphocyte development and maturation.^14^^,^^15^ In addition to the known outcomes of transcription factors on stem cell commitment toward a lineage, other factors such as lncRNAs may be the key to mediate the maintenance of lymphocyte subsets using their lncRNA expression profiles.^15^ Aberrant expression of lncRNAs can change the pathway of growth and apoptosis in cells.^16^^,^^17^ Consequently, such changes lead to the transformation of normal cells into cancerous cells such as lymphomas.^16^^,^^17^ The findings of de novo lncRNAs is challenging due to the low expression and statistical complexity of discovery. However, there are a limited number of studies about the dysregulation of lncRNAs in lymphomas. In this review, we summarize the current knowledge on the function of lncRNAs in lymphoma pathogenesis, their molecular role, and possible clinical benefit.

## The role of lncRNAs in miRNAs synthesis in lymphomas

**Figure 3:**
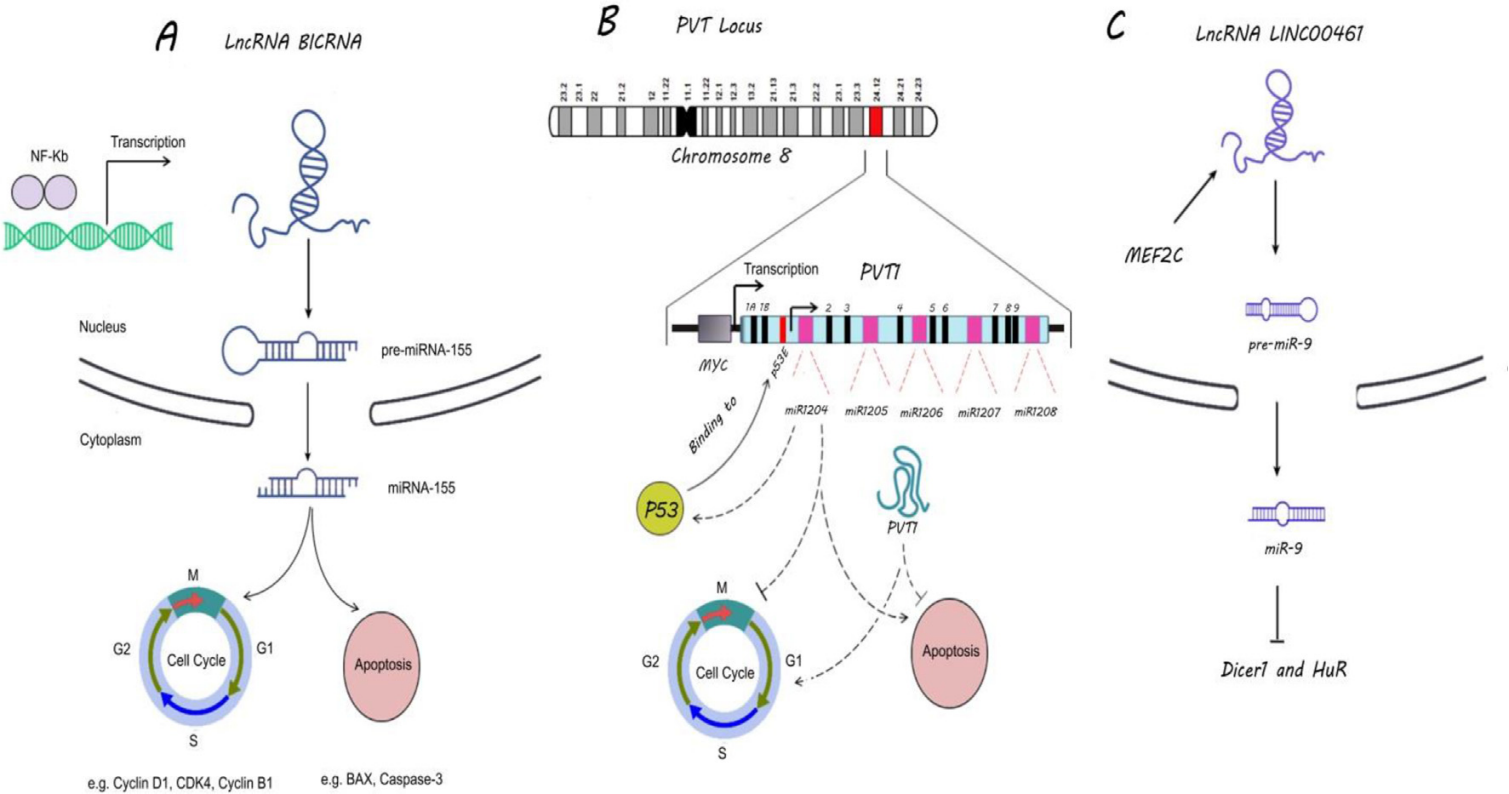
The role of lncRNAs in miRNAs synthesis. **(A)** Interaction between NF-κB/BIC and RNA/miR-155 in lymphoma cells has been shown. NF-κB leads to the increased transcription of lncRNA BIC RNA. This lncRNA can act as pre-miR-155, which actives the cell cycle and inhibits apoptosis. **(B)***PVT1* locus is located on chromosome 8q24.21 and encodes lncRNA PVT1 and a wide variety of non-coding microRNAs, including miR-1204, -1205, -1206, -1207, and -1208. Myc leads to the elevated expression of lncRNA PVT1 and other miRNAs. Furthermore, p53 has a positive effect on the transcription of miR-1204. There is positive feedback between p53 and miR-1204, which inhibits the cell cycle and induces apoptosis. However, lncRNA PVT has an oncogenic role in lymphoma cells. **(C)** There is a network between lncRNA LINC00461, MEF2C, and miR-9. LncRNA LINC00461 can act as pre-miR9. MiR-9 actively participates in lymphoma pathogenesis by targeting *DICER1* and HuR. NF-κB, nuclear factor kappa-light-chain-enhancer of activated B cells; PVT1, plasmacytoma variant translocation 1 (PVT1); MEF2C, myocyte-specific enhancer factor 2C.

MiRNAs are non-coding RNAs, which have a critical function in the regulation of mRNA stability and translation.^18^ It has been demonstrated that lncRNAs can function as miRNA precursors.^19^ MiR-155 plays an important role in the pathogenesis of B cell lymphoma by targeting the sequences of *PU.1* and CCAAT/enhancer-binding protein β (*C/EBPβ*).^20^ MiR-155 increases cell cycle-correlated proteins such as cyclin-dependent kinase 4 (CDK4), cyclin D1, and B1.^21^ Additionally, it can reduce apoptosis-correlated proteins, including B-cell lymphoma 2-associated X (BAX) and caspase-3 activities.^21^ Peggy et al showed the generation of pre-miR-155 within the nucleus by processing the intron-free BIC RNA.^19^ BIC RNA and miR-155 are highly expressed in DLBCL, HL, and more indolent lymphomas.^19^^,^^22^ The comparison between GCB-like and ABC-like DLBCL indicated more expression of BIC RNA and miR-155 in ABC-like phenotype.^19^ A nuclear factor kappa light chain enhancer of activated B cells (NF-κB) binding site is in the promoter region of the *BIC* gene.^23^ Induction of NF-κB can enhance the expression of BIC RNA and miR-155.^23^ It seems that there is a network between NF-κB, BIC RNA, and miR-155 in lymphoma cells (Fig. 3A).^22^^,^^23^

Plasmacytoma variant translocation 1 (*PVT1*) locus is located downstream of the myelocytomatosis (*Myc*) locus on chromosome 8q24.21 and encodes lncRNA PVT1 and a wide variety of microRNAs with suspected oncogenic properties, including miR-1204, -1205, -1206, -1207, and -1208.^24^ LncRNA PVT1 is co-increased in *Myc*-copy-increase malignancies such as BL, T-lineage lymphoma, and HL.^24^^,^^25^ Translocation breakpoints within either *PVT1* locus or *Myc* are the characteristic lesions associated with BL.^26^ Survival roles of lncRNA PVT1 in malignant cells have been demonstrated, but some of the miRNAs such as miR-1207 and miR-1204 act as a tumor suppressor.^27^ P53 as an apoptotic-induced protein induces the expression of miR-1204 by binding to exon 1B of *PVT1* locus (Fig. 3B).^27^ Interestingly, miR-1204 also improves the p53 protein levels and indicates positive feedback to increase p53 activity.^27^ Wang et al showed that lncRNA PVT1 induces proliferation, invasion, and angiogenesis of cancer by sponging miR-1204 and miR-1207.^28^ Therefore, the stimulation of *PVT1* locus is like a double-edged sword in which miR-1204 and miR-1207 might elevate cell death, whereas the lncRNA PVT1 prevents cell death (Fig. 3B).^27^, ^28^, ^29^

MiR-9 affects pathological mechanisms underlying HL by targeting *Dicer-1* and *HuR*.^30^ Suppression of miR-9 can reduce the outgrowth and the ability of HL cells to secrete cytokines.^30^ Transcription factor myocyte-specific enhancer factor 2C (MEF2C) induces the expression of miR-9-2 through binding to a site on the miR-9-2 promoter in the last exon of *LINC00461* gene (Fig. 3C).^31^ MEF2C is mandatory in response to B-cell receptor (BCR) stimulation.^32^ It has been shown that lncRNA LINC00461 knockdown decreases the expression levels of miR-9 and even MEF2C.^33^ How LINC00461, MEF2C, and miR-9 regulate each other's expression in lymphomas remains unclear.

## The function of lncRNAs as competitive endogenous RNAs (ceRNAs) in lymphomas

LncRNAs may compete with mRNAs for binding to miRNAs.^34^ Such a network between lncRNA and miRNA decreases miRNAs in the cytoplasm, leading to inhibition of miRNA binding to mRNA and enhancement of mRNA stability.^34^

*BRAFP1* is located on chromosome X and acts as a competitive endogenous RNA.^35^ It can increase BRAF mRNA stability through competition in binding to sequester endogenous miR-30a,-134,-182, -543, -653, and -876.^35^ BRAF activates tumorigenesis by mitogen-activated protein kinase (MAPK) signaling pathway.^35^ Therefore, it is considered that increasing X dosage in DLBCL contributes to the development and progression of cancer by overexpression of *BRAFP1*.^35^

MiR-135b-5p is associated with tumor growth and cancer development.^36^^,^^37^ Zhao et al demonstrated a complementary sequence of miR-135b-5p in lncRNA SMAD5 antisense RNA 1 (SMAD5-AS1), which reduces the lymphoma growth by absorbing miR-135b-5p.^38^ In non-small cell lung cancer (NSCLC), lncRNA growth arrest-specific 5 (GAS5) inhibits miR-135b-5p and enhances radiosensitivity.^39^ Despite the expression of lncRNA GAS5 in lymphomas, the interaction of this lncRNA with miR-135b-5p has not been determined yet.^39^

LncRNA HOX transcript antisense RNA (HOTAIR) suppresses miR-148b in lymphoma cells.^40^ High expression of HOTAIR influences the growth of lymphoma cells by downregulation of miR-148b expression.^40^ Also, lncRNA HOTAIR can repress other miRNAs such as miR-205, -141, and -130a in several cancer cells, including bladder, renal, and gallbladder cancer cells.^41^, ^42^, ^43^ However, the effect of lnRNA HOTAIR on these miRNAs in lymphomas is unclear.

## Interaction between lncRNAs and polycomb group in lymphomas

**Figure 4:**
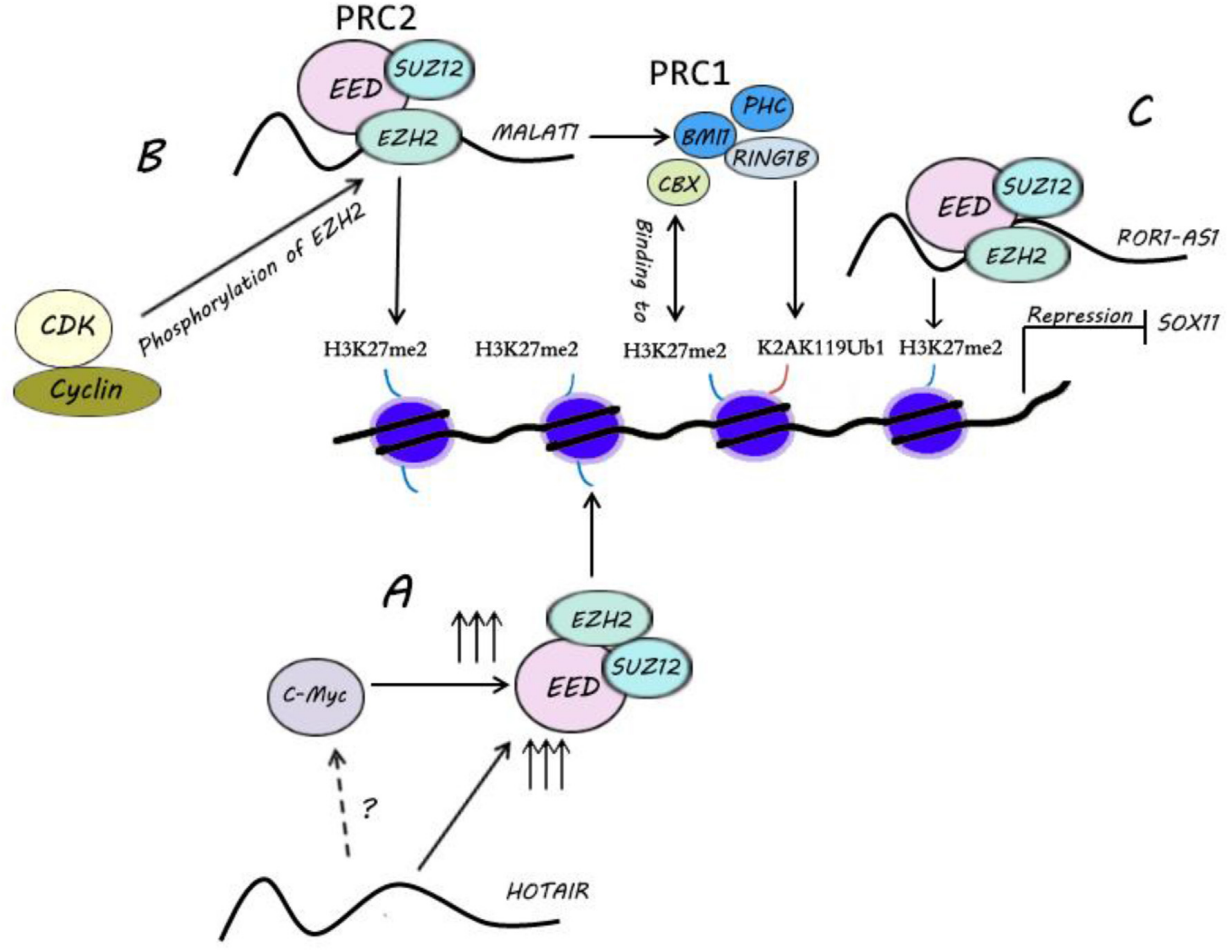
Simplified model of PcG regulation of transcription. The complex of PRC2 is recruited to chromatin, and the EZH2 protein trimethylates histone H3 at lysine 27. Then, this mark acts as a binding site for the CBX proteins of the PRC1 complex. The interaction of CBX proteins with H3K27me3 anchors the complex of PRC1 to chromatin. RING1B is an E3 ubiquitin ligase and catalyzes H2AK199ub formation. Finally, these activities lead to folding and compaction chromatin and transcriptional repression. **(A)** HOTAIR is involved in inducing H3K27me3 levels by recruiting PRC2 proteins, including EZH2, SUZ12, and EED. The possible interaction of HOTAIR with c-Myc in recruiting the PRC2 complex is unknown. C-Myc leads to the induction of PRC2 proteins. **(B)** CDK1 and CDK2 induce the phosphorylation of EZH2, resulting in increased binding to lncRNA MALAT1. Bmi1 does not bind directly to lncRNA MALAT1, but lncRNA MALAT1 induces Bmi1 activation through recruiting SUZ12 and EZH2. **(C)** ROR1-AS1 physically interacts with the PRC2 subunits (EED, SUZ12, and EZH2) and suppresses SOX11 expression in MCL cells. PcG, polycomb group; PRC, polycomb repressive complex; EZH2, enhancer of Zeste homolog 2, CBX, chromobox homolog; HOTAIR, HOX transcript antisense RNA; SUZ12, suppressor of Zeste 12 homolog; CDK, cyclin-dependent kinase; Bmi1: B-lymphoma Mo-MLV insertion region1 homolog; MALAT1, metastasis associated lung adenocarcinoma transcript 1; SOX11, SRY-related HMG-Box 11.

Polycomb group (PcG) proteins maintain gene expression patterns of different cells by regulating chromatin structure.^44^ Two main PcG complexes exist in mammals, including the polycomb repressive complex 1 (PRC1) and PRC2 (Fig. 4).^44^ PRC1 consists of B lymphoma Mo-MLV insertion region1 homolog (Bmi1), PHC, chromobox (CBX), and RING1B. The PRC2 complex has three subunits; enhancer of zeste homolog 2 (EZH2), suppressor of zeste 12 homolog (SUZ12), and EED1.^44^ Generally, PRC2 is associated with the trimethylation of histone H3 at lysine 27 (H3K27me3), and PRC1 interferes with the genome regions through H3K27me3 (Fig. 4).^45^ PRC2 has a crucial role in B cell development and rearrangement of the immunoglobulin chain gene.^46^ It has been demonstrated that EZH2 is the mediator of histone H3 methylation, which controls immunoglobulin heavy chain gene rearrangement during early murine B cell development.^46^ Elevated expression of EZH2 is associated with enhanced malignancy and poor prognosis in cancers.^47^ Inhibition of EZH2 methyltransferase in DLBCL suppresses global H3K27me3 levels and subsequently reactivates silenced PRC2 target genes.^48^ Therefore, increased H3K27me3 levels can be an inferior overall survival in lymphoma patients.^49^ Some lncRNAs have been known to regulate gene expression through a mechanism involving interaction with the PRC pathway.^50^, ^51^, ^52^, ^53^ Studies on over 3300 lncRNAs revealed that 20% of lncRNAs exerts as binding partners for PRC2 in various cells.^50^

About 24% of DLBCL patients have increased lncRNA HOTAIR expression levels, and high expression of EZH2 is more frequent in HOTAIR high than HOTAIR low cases.^52^ HOTAIR promotes H3K27me3 levels by recruiting PRC2 proteins such as EZH2, SUZ12, and EED (Fig. 4A).^52^ C-Myc interacts with EZH2 and SUZ12/EED, and this complex stimulates the histone modification of H3K27me3 on the promoter of target genes.^52^^,^^54^ Further studies have implicated that c-Myc and EZH2 induce each other.^55^^,^^56^ The close association of PRC2 with HOTAIR and c-Myc has not been proven in hematologic malignancies.

Metastasis associated lung adenocarcinoma transcript 1 (MALAT1), which is known as nuclear enriched abundant transcript 2 (NEAT2) is an 8.7 kb transcript, located on chromosome 11q13, a site in the adjacency of translocation (11; 14).^57^ There is a high affinity of binding between PRC2 and lncRNA MALAT1 in MCL and natural killer (NK)/T cell lymphoma (Fig. 4B).^53^^,^^58^ In MCL, CDK1 and 2 induce the phosphorylation of EZH2 at threonine 350 (T350), which increases the binding to lncRNA MALAT1 (Fig. 4B).^53^ It has been shown that MALAT1-induced H3K27me3 directly promotes the activation of Bmi1.^58^

Receptor tyrosine kinase-like orphan receptor 1 (ROR)1-AS1 is up-regulated in most of the MCL patients, but its expression is low in MCL cell lines.^59^ ROR1-AS1 physically interacts with the PRC2 subunits (EED, SUZ12, and EZH2) and suppresses SRY-related HMG-box 11 expression of (*SOX11*) gene in MCL (Fig. 4C).^59^ Transcription factor SOX11 can be a specific biomarker of MCL.^60^ The prognostic value of SOX11 in a population-based study of 186 cases of MCL patients showed that 88% of indolent MCL patients with less frequent B symptoms express SOX11 transcription factor.^60^ As a result, most indolent MCL patients are SOX11 positive, but SOX11 cannot predict an indolent disease course.^60^ High expression level of antisense non-coding RNA in the INK4 locus (ANRIL) has been observed in adult T cell leukemia (ATL) samples.^61^ ANRIL is associated with EZH2 and induces the NF-κB signaling pathway in ATL cells.^61^ Complex of lncRNA ANRIL and EZH2 increase P65 binding capability to target genes.^61^

## The role of lncRNAs in chemotherapy response of lymphoma patients

Chemotherapy and radiotherapy still comprise the basis of treatment strategies in lymphomas.^62^^,^^63^ The standard therapy for NHL includes doxorubicin, cyclophosphamide, vincristine, and prednisolone.^62^ Unfortunately, a significant population of NHL patients undergoes relapse, resulting in disappointing 3-year overall survival (OS) of about 30%.^64^ Higher doses of chemotherapy to relapsed individuals result in severe adverse outcomes, but unfortunately, the response in relapsed patients is about 30%.^64^ Relapsed lymphomas can be due to the appearance of subpopulations of drug-resistant cancer cells, which is the underlying cause of failure in the standard therapies.^65^ Fundamental genetic changes and abnormalities in lncRNAs of malignant cells can also explain the failure of combination chemotherapy in lymphomas.^66^ Chemotherapy-resistant cell lines express a high level of lncRNA MALAT1.^67^ MALAT1 induces chemoresistance in DLBCL by inhibiting the autophagy signaling pathway.^67^ MALAT1 increases P62, a classical receptor of autophagy, and suppresses the proteins of light chain 3 (LC3)-II and LC3-I.^67^ During stress conditions such as chemotherapy, damaged proteins are ubiquitinated and degraded by the proteasome and autophagy.^68^ P62 binds to polyubiquitinated proteins to direct these proteins to the pathway of autophagy.^68^ Eventually, this complex binds to Atg8/LC3 on the autophagosome membrane for degradation.^68^ The inability of autophagy to omit p62 leads to the resistance of lymphoma cells to chemotherapy.^68^ However, the effect of lncRNA MALAT1 on other autophagy proteins like Beclin1 is unclear.

Some lncRNAs cause sensitivity to chemotherapeutic agents in lymphoma cells.^69^^,^^70^ Maternally expressed gene 3 (MEG3) acts as a favorable prognostic factor in T-lymphoblastic lymphoma (T-LBL) and leads to the sensitivity of T-LBL to chemotherapy.^69^ In T-LBL cells with MEG3 overexpression, the phosphorylation of PI3K/Akt and mammalian target of rapamycin (mTOR) is reduced.^69^ PI3K/mTOR signaling pathway elevates the expression of P-glycoprotein (P-gp) and leads to the occurrence of tumor MDR.^69^ It has been demonstrated that a major part of the effect of mTOR inhibitors such as rapamycin, everolimus, and temsirolimus on MCL cells is regulated by lncRNA GAS5.^70^ Inhibition of mTOR reduces the translation of several groups of RNAs, including 5′TOP transcripts such as GAS5 and mRNAs encoding cell cycle regulators like cyclin D1.^70^ Therefore, lncRNA GAS5 can act as an effector of the mTOR pathway and its transcripts are stabilized through mTOR inhibition.^70^

Other lncRNAs as biomarkers for the good prognosis of response to chemotherapy are lncRNA NONHSAG026900 and paternally expressed 10 (PEG10).^71^ DLBCL patients with high-value expression of NONHSAG026900 have a better response to CHOP compared to those with low values.^72^ The expression level of NONHSAG026900 is higher in patients with GCB-DLBCL than non-GCB-DLBCL patients, and the first group has a more favorable outcome compared to the second one.^72^ The expression level of PEG10 can affect chemotherapy-induced apoptosis.^73^ Findings have shown that downregulation of PEG10 by siRNAs results in an increased apoptosis rate of 30% in BL patients treated with 5- fludarabine (FU).^73^ Thus, elucidation of lncRNA roles in drug-resistant lymphomas can improve the efficacy of current therapeutic strategies.

## The effect of lncRNAs on survival and apoptosis of lymphoma cells

The apoptotic and proliferative indices of lymphomas are beneficial prognostic indicators, which provide independent prognostic information from other clinical and histological variables.^74^ Proliferative parameters alone do not mean an increase in cell growth.^74^ A high cell production rate can be compensated through high apoptosis.^74^ More than 200 lncRNAs are in proximity to genes that can involve cell growth and cell death simultaneously, and may disturb the balance of cell proliferation and apoptosis.^75^

**Figure 5:**
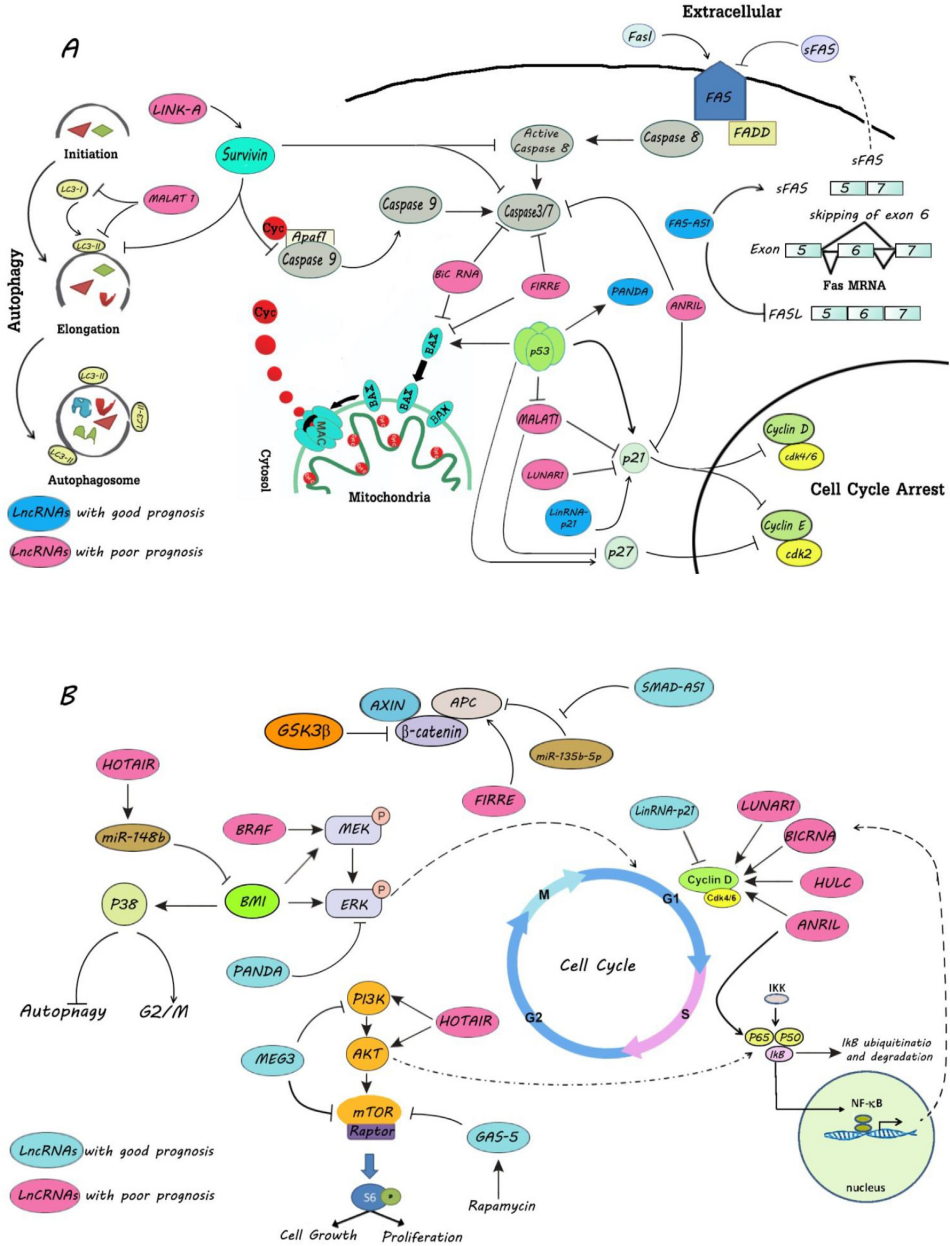
The effect of lncRNA on proliferation and apoptosis. **(A)** LncRNAs can affect the proliferation of lymphoma cells by p38 MAPKs signaling pathway (e.g., HOTAIR), MAPK/ERK pathway (e.g., HOTAIR and PANDA), mTOR pathway (e.g., MEG3, HOTAIR, and GAS-5), Wnt signaling pathway (e.g., SMAD5-AS1 and FIRRE), cyclin D (e.g., LUNAR1, BICRNA, HULC, ANRIL, and LincRNA-p21), and NF-κB signaling pathway (e.g., ANRIL). **(B)** LncRNAs can affect the apoptosis of lymphoma cells by autophagy pathway (e.g., MALAT1), caspase pathway (e.g., Link-A, BIC RNA, FIRRE, and ANRIL), p53 pathway (e.g., MALAT1, LUNAR1, LincRNA-p21, and PANDA), and Fas ligand pathway (e.g., FAS-AS1). MAPK, mitogen-activated protein kinase; HOTAIR, HOX transcript antisense RNA; ERK; extracellular-signal-regulated kinase; HOTAIR, HOX transcript antisense RNA; PANDA, p21-associated ncRNA DNA damage–activated; mTOR, mammalian target of rapamycin; MEG3, maternally expressed gene 3; GAS5, growth arrest-specific 5; ANRIL, antisense non-coding RNA in the INK4 locus; MALAT1, metastasis associated lung adenocarcinoma transcript 1; LUNAR1, leukemia-associated non-coding IGF1R activator RNA 1.

LncRNA MALAT1 increases cell proliferation and suppresses the apoptotic percentage in MCL and DLBCL patients (Fig. 5A, B).^53^^,^^67^ It has been shown that there is a two-way regulatory communication of lncRNA MALAT1 and p53 in lymphoma cells.^53^^,^^76^ There are two p53 binding sites on the promoter region of lncRNA *MALAT1* gene.^76^ Overexpression of p53 decreases the expression of MALAT1.^76^ Hence, p53 regulates the inhibitory effect of MALAT1 on p21 and p27 (Fig. 5A).^78^ On the other hand, MALAT1 suppresses p53 by increasing H3K27me3 on the *TP53* promoter.^53^ Moreover, MALAT1 prevents the suppression of CDKs by an EZH2-associated mechanism in MCL.^53^ Nevertheless, p53 stimulates the expression of some lncRNAs like P21-associated ncRNA DNA damage -activated (PANDA) through interacting with the promoter of *PANDA* gene.^79^ LncRNA PANDA suppresses cell proliferation by inhibiting proteins involved in the MAPK/ERK signaling pathway (Fig. 5B).^79^ The downregulation of serum mRNA of p53 and PANDA have been illustrated in DLBCL patients.^79^

The cyclin D1 proto-oncogene acts as an essential accelerator of G1 to S phase progression in different cell types.^80^ Cyclin D1 appears to be a target for a high number of lncRNAs (Fig. 5A, B).^81^^,^^82^ Hepatocellular carcinoma up-regulated long non-coding RNA (HULC) could facilitate cell proliferation by increased expression of cyclin D1 in DLBCL.^81^ Furthermore, lncRNA leukemia-associated non-coding IGF1R activator RNA 1 (LUNAR1) leads to increased cell proliferation and decreased apoptosis through cyclin D1 and p21 in DLBCL, respectively (Fig. 5A, B).^82^ LncRNA ANRIL plays a key role in maintaining the proliferation of ATL cells.^61^ ANRIL increases cyclin D1 and E1 expression and suppresses cleaved caspase-3, caspase-7, and poly (ADP-ribose) polymerase (PARP).^61^ Moreover, p21/CDKN1A is a novel target of lncRNA ANRIL in ATL and its expression is decreased by ANRI.^61^

Survivin is a member of the inhibitor of apoptosis (IAP) gene family, and its expression is observed in growing cells and tumor cells.^83^ Survivin has an important role in cancer cell survival networks by inhibiting caspase-9, -8, -7, and excessive autophagy proteins (Fig. 5B).^84^^,^^85^ The levels of long intergenic non-coding RNA for kinase activation (LINK-A) and survivin are significantly high in plasma of patients with MCL.^86^ In fact, LINK-A promotes lymphoma cell survival by increasing survivin expression (Fig. 5A).^86^

C-Myc belongs to the myc family and acts as a central oncogenic switch in various human malignancies.^87^ In addition to c-Myc, N-Myc and L-Myc have neoplastic potential role.^87^ The improper expression of c-Myc is thought to be the central oncogenic switch that promotes the development of malignancies.^87^^,^^88^

LncRNA ENSG00000253716, which is known as MINCR has a strong positive correlation with the expression level of Myc in BL and other Myc-positive lymphomas.^89^ Myc binding regions are present around TSS of *MINCR*, suggesting that MINCR acts as a regulator of the Myc transcriptional program.^89^ LncRNA MINCR knockdown is associated with impairment in the expression of cell cycle genes in BL cells.^89^ Interestingly, there are Myc binding sites in the promoters of cell cycle genes.^89^ Hence, MINCR knockdown decreases Myc binding to the promoters of cell cycle genes such as aurora kinase (*AURK*) *A* and *B*.^89^ AURK proteins are serine/threonine kinases, which are overexpressed in many tumors.^90^ They lead to polyploidy and multinucleation of cells.^90^ The main role of AuroraA is the coordination of centrosome maturation and chromosome separation.^90^ AuroraB results in the phosphorylation of chromatin proteins to aid in mitotic chromosome condensation.^90^ LncRNA FIRRE is another lncRNA that is transcriptionally activated by Myc.^91^ It has been observed that Myc binding sites are on the promoter of *FIRRE*.^91^ Myc-induced expression of lncRNA FIRRE suppresses apoptosis by decreasing caspase-3 and BAX (Fig. 5A).^91^ Furthermore, FIRRE stimulates Wnt/β-catenin signaling pathway through increasing nuclear translocation of β-catenin to facilitate DLBCL proliferation.^91^ However, the function of some lncRNAs, which are recruited by Myc like NAALADL2-AS2 has not been demonstrated.^92^

LncRNA SMAD5-AS1 related to DLBCL could lead to the activation of Wnt/β-catenin signaling pathway and facilitates β-catenin expression in the nucleus by increased expression of anaphase-promoting complex subunit (APC) (Fig. 4).^38^ The *APC* gene is a direct target of miR-135b-5p, and SMAD5-AS1 increases expression of APC significantly by inhibiting miR-135b-5p expression (Fig. 5B).^38^ In fact, the SMAD5-AS1/miR-135b-5p axis activates Wnt/β-catenin pathway through the specific meditation on APC (Fig. 5B).^38^

Bmi1 has an oncogenic role in lymphomas development through the phosphorylation of p38 MAPKs and ERK (Fig. 4).^93^ Furthermore, the upregulation of Bmi1 leads to the repression of cell-cycle regulators like p16INK4a/p19ARF and emersion of lymphoma in Bmi1 transgenic mice.^94^ LncRNA HOTAIR promotes Bmi1 expression in lymphoma cells by inhibiting the regulatory effect of miR-148b on Bmi1.^93^ Also, HOTAIR stimulates the phosphorylation of PI3K/Akt and NF-κB, which leads to increased cell proliferation (Fig. 5B).^93^

Some lncRNAs act as an anti-oncogenic factor in lymphomas.^95^ LncRNA lincRNA-p21 arrests growth and cell cycle progression of lymphoma cells by downregulating cyclin D1, CDK4, and upregulating the expression of p21 (Fig. 5A, B).^96^ In lymphomas, soluble Fas receptor (sFas) inhibits apoptosis by sequestering Fas ligand.^97^ SFas is produced by skipping of exon 6 of Fas mRNA maturation.^97^ Alternative splicing of Fas mRNA is reversely regulated by lncRNA FAS-AS1.^97^ Levels of FAS-AS1 correlate inversely with the production of sFas, and FAS-AS1 binding to the RNA binding motif protein 5 (RBM5) inhibits RBM5-mediated exon 6 skipping.^97^ However, EZH2 hyper-methylates the promoter of FAS-AS1 in lymphoma cells and suppresses the FAS-AS1 expression.^97^

LncRNA ROR1-AS1 induces the proliferation of MCL cells.^59^ LncRNA ROR1-AS1 is also induced in B cells treated with CD40L and IgM.^59^ These findings demonstrated that lncRNA ROR1-AS1 is involved in receptor signaling of B lymphocytes. However, the interaction of ROR1-AS1 with proteins involved in the cell cycle is not clear. Moreover, LncRNA PEG1 increases proliferation and decreases apoptosis in DLBCL, however, the functional mechanism of lncRNA PEG1 is still unknown.^98^

## The effect of lncRNAs on invasion of lymphoma cells

Recent data have provided new insights into the mechanism of lncRNAs, which are related to the invasion of cancers. LncRNA LINC01013, as a metastatic marker, contributes to the induction of anaplastic large cell lymphoma (ALCL) cell invasion.^99^ This lncRNA plays a potential role in ALCL progression by the stimulation of the snail-fibronectin cascade.^99^ The transcription factor snail is a regulator of epithelial-mesenchymal transitions (EMT) and plays a crucial role in metastatic dissemination.^100^ Snail suppresses the expression of E-cadherin strongly.^100^

Degradation of extracellular matrix (ECM) is the hallmark of migration and invasion of cancer cells.^101^ The matrix metalloproteinase (MMP), including MMP-2 and MMP-9 are critical enzymes, which can degrade all of the components of ECM.^101^ Upregulation of MMP-2 and MMP-9 contributes to high invasion and infiltration of lymphoma cells.^101^ There is an association between increased expression of lncRNA PEG10 and the invasion abilities of lymphoma cells.^73^ PEG10 promotes the migration and invasion of lymphoma cells by elevating MMP-2 and MMP-9.^73^

On the other hand, some lncRNAs act as a suppressor of the migration of lymphoma cells.^69^ The overexpression of lncRNA MEG3 increases the epithelial marker E-cadherin and decreases mesenchymal marker N-cadherin.^69^ Further, MEG3 suppresses the expression of vimentin and Snail.^69^ It suggests that lymphoma cells with decreased expression of lncRNA MEG3 are more predisposed to EMT and potentially associated with cell migration and invasion.^69^

## Correlation of lncRNAs with prognosis in lymphoma patients

International Prognostic Index (IPI) for patients with NHL is based on pretreatment clinical characteristics, including Ann Arbor stage, lactate dehydrogenase (LDH), performance status, and the number of extranodal disease sites.^102^ Therefore, four groups of patients were identified, including low risk (IPI = 0 or 1), low-intermediate risk (IPI = 2), high-intermediate risk (IPI = 3), and high risk (IPI = 4 or 5).^103^ Novel prognostic approaches allow the identification of high-risk groups and might provide opportunities to select specific treatment approaches.^103^ We summarize the lncRNAs that can be effective in diagnostic and prognostic markers in lymphomas (Table 1).

**Table 1:** The role of lncRNAs in lymphoma patients.

LncRNA	Location	Study	Prognosis	Reference
ANRIL	Chr9p21.3	HTLV-1-infected T-cell lines and HTLV-1-negative T-cell lines	Poor	^61^
		6 cases of ATL		
BIC RNA	Chr21q21	HL cell lines (HDLM2, L428, KMH2, L591, and L1236)	Poor	^19^
		GC-related DLBCL line OCI-Ly1 non-GC DLBCL lines (OCI-Ly8, OCI-Ly3)		
		Tissue samples of DLBCL		
		cHL cell lines, NHL cell lines	Poor	^22^
		Tissue samples of HL and various NHL cases		
BRAFP	ChrXq13.3	SU-DHL-4, SU-DHL-8, Karpas422, OCI-Ly7, Toledo, OCI-Ly1, and OCI-Ly18	Poor	^35^
ENSG00000253716 (MINCR)	Chr8q24.3	Cell line expressing the MycER fusion protein (hT-RPE-MycER)	Poor	^89^
FAS-antisense 1	Chr10q23.31	Granta-519 cells	Favorable	^97^
Firre	ChrXq26.2	DLBCL cell lines (U2932, SU-DHL-6, SU-DHL-4, OCL-LY-7, OCL-LY-10)	Poor	^91^
		70 cases of DLBCL		
FLJ42351	Chr2q14.1	5 cases of cHL	Poor	^14^
GAS5	Chr1q25.1	MCL lines (Jeki-I and Z-138)	Favorable	^70^
HULC	Chr6p24.3	142 cases of DLBCL	Poor	^81^
HOTAIR	Chr12q13.13	164 cases of DLBCL	Poor	^52^
		DLBCL cell lines (RCK-8, OCL-LY-10, OCL-LY-7, SU-DHL-6 and SU-DHL-4)	Poor	^104^
		50 cases of DLBCL		
		46 cases of lymphoma None of the patients received chemotherapy or radiotherapy Human lymphoma cell lines (Raji and U937 cells)	Poor	^93^
LINC01013	Chr6q23.2	ALCL cell lines (SR-786, KARPAS-299, and Matrigel-selected KARPAS-invasive human ALK(+))	Poor	^99^
LincRNA-p21	Chr17p13.1	105 cases of DLBCL DLBCL cell lines (SU-DHL-2, OCI-LY-3, OCILY-10, SU-DHL-4 and OCI-LY-7)	Favorable	^95^
LUNAR1	Chr15q26.3	87 cases of DLBCL	Poor	^82^
LINC00461	Chr5q14.3	5 cases of cHL	Poor	^14^
LINC00116	Chr2q13		Poor	
LINK-A (LOC339535/NR_015407)	Chr1q43	MCL cell lines (JVM-2 and Z-138) 36 cases of MCL	Poor	^86^
MALAT1	Chr11q13	Cell lines (Mino and Jeko-1) 40 cases of MCL	Poor	^53^
		Cell Lines (Farage, Pfeiffer, Raji, Daud, Ly1, Ly3, Ly8, and Ly10)	Poor	^67^
		167 cases of NK/T-cell lymphoma	Poor	^58^
MEG3	Chr14q32.2	T-LBL cell lines (Jurkat and SUP T1)	Favorable	^69^
NAALADL2-AS2	Chr3q26.31	ABC-like DLBCL cell lines (OCI-ly10 and U-2932), 3 GCB-like DLBCL cell lines (OCI-ly19, SU-DHL-4, and DB)	Poor	^92^
NONHSAG026900	Chr1p36.22	Microarray data sets from the GEO database consisting DLBCL samples	Favorable	^72^
PANDA	Chr6p21.1	DLBCL cell lines (U2932, SUDHL-6, SUDHL-3, OCI-Ly3, and OCI-Ly8)	Favorable	^79^
		114 cases of DLBCL		
PEG10	Chr7q21	107 cases of DLBCL	Poor	^98^
		BL cell line (Raji)	Poor	^101^
PVT1	Chr8q24.21		poor	^24^
ROR1-AS1	Chr1p31.1	MCL cell lines Mino, Granta, JVM2 and Z138	Poor	^59^
		5 cases of MCL		
SMAD5-AS1	Chr5q31.1	GCB DLBCL cell lines (TMD8 and U2932), ABC DLBCL cell line (OCI-Ly3), FL cell line (WSU-FSCCL), MCL cell line (JeKo-1), cHL cell line (L428), and Burkitt's lymphoma cell line (Raji)	Favorable	^38^
		11 cases of DLBCL		
SubSigLnc-17∗	–	GEO, including GSE31312 cohort (*N* = 426), GSE10846 (*N* = 350)cohort and GSE4475 cohort (*N* = 129)	Favorable/poor	^71^

LncRNA HOTAIR is remarkably associated with increased tumor volumes, IPI scores, B symptoms, and clinical stage.^104^ Increased expression of HOTAIR predicts a poor prognosis in DLCBCL patients, whereas the lower HOTAIR possesses higher overall survival probabilities.^93^ LncRNA PEG10 is significantly correlated with IPI score, B symptoms, and OS, implicating that PEG10 could be a promising biomarker in DLBCL.^73^ Furthermore, DLBCL patients with Ann Arbor stages (III-IV) and high IPI score have elevated expression of HULC.^81^

Investigating lncRNA NONHSAG026900 expression and clinical features in 170 patients with DLBCL exhibited that this lncRNA could act as the predictive power of IPI.^72^ 5-year OS rates in patients with a low value of NONHSAG026900 were poorer than those with high value.^72^ Moreover, patients with high expression levels of lincRNA-p21 had a significantly higher survival rate than those with low levels, suggesting the anti-oncogenic role of lincRNA-p21 in lymphoma progression.^95^

LncRNA PANDA is remarkably associated with Ann Arbor stages, B symptoms, and IPI, while there is no correlation between the expression of PANDA and other pathological factors, including age, gender, performance status, and subtypes in patients.^79^ In DLBCL patients, lncRNA PANDA is significantly associated with a good prognosis.^79^

MALAT1 is correlated with poor prognosis in T and NK cell, MCL, and DLBCL lymphoma.^53^^,^^58^ Correlation between the expression of MALAT1 in NK/T-cell lymphoma and clinicopathologic variables showed that patients with high expression of MALAT1 had low OS.^58^

Analysis of lncRNA profile in DLBCL patients showed a set of six lncRNAs, including SACS-AS1, MME-AS1, CSMD2-AS1, RP11-360F5.1, RP11-25K19.1, and CTC-467M3.1, which are substantially correlated with the prognosis of DLBCL patients.^105^ These six-lncRNAs signature could estimate overall survival in DLBCL patients with the same variables of IPI, providing additional information beyond the conventional IPI system.^105^ Patients were assigned to two groups based on six-lncRNAs expression, including the high-risk group and low-risk group.^105^ DLBCL patients in the low-risk group showed a better overall 5- and 10-year relative survival rate in comparison with those who were in the high-risk group.^105^ Further analysis of prognostic values of these six-lncRNAs in the additional independent patient dataset from Visco's study showed that patients in high-risk groups had remarkably shorter OS than those belonging to low-risk groups.^105^ However, further studies are required to uncover the molecular function of these lncRNAs and other prognostic lncRNAs in DLBCL.

## Undetermined significance of LncRNAs in lymphomas

The precise function of some lncRNAs has not been fully characterized, despite the increase and decrease of these molecules in lymphoma cells. Verma et al found 2632 novel lncRNAs in DLBCL by investigating RNA-seq data from primary DLBCL samples.^106^ Two-thirds of these novel lncRNAs were not expressed in normal B lymphocytes.^106^ A direct comparison of DLBLC cell lines with normal B cells showed substantial levels of differential expression for 1053 lncRNAs (fold change > 1.5, FDR < 0.05).^92^ 416 lncRNAs were up-regulated in DLBCL cell lines whereas 637 lncRNAs were down-regulated.^92^ Moreover, the expression pattern of lncRNAs in cHL and normal B lymphocytes indicated substantial differential expression for 475 lncRNAs loci, which 75% of these lncRNAs are down-regulated in cHL.^14^

Genome-wide analysis of lncRNA expression profiles in DLBCL patients revealed 17 lncRNAs based signatures (SubSigLnc-17).^71^ These SubSigLnc-17 correctly classified DLBCL patients to ABC-like and GCB-like with an accuracy of 91.1%.^71^ In the predicted GCB-like group, the overall survival rate was significantly higher than the ABC-like group.^71^ The function of SubSigLnc-17 in the cell cycle and apoptosis in GCB and ABC subtypes is not known.^71^ Wang et al revealed potential lncRNAs, which are distinctly expressed in DLBCL patients by Hiseq array in the discovery phase.^79^ They recognized 546 lncRNAs that were differentially expressed, including FIRRE, PEG10, and LUNAR1.^79^ However, in contrast to previous studies, there is no significant pathogenesis role for FIRRE, PEG10 and LUNAR1 in DLBCL patients.^79^ Expression levels of lncRNAs may be different in various samples and different diseases. So, different clinical materials should be used to ensure that the chosen lncRNAs are qualified for using in clinical prognosis.

The study on aberrantly expressed lncRNAs in ALCL identified five lncRNAs, which were highly expressed in ALCL, including ~5, ~10, ~15, ~17, and ~19-fold for CACNA1G-AS1, BMS1P20, RNF144A-AS1, LINC01013, and MIR503HG, respectively.^99^ Among these, the function of LINC01013 has been validated in tumor cell invasion, however, no recent studies have reported an interaction between other lncRNAs and progression of ALCL.^99^ Pan et al identified 189 differentially expressed lncRNAs in FL compared to reactive lymphatic nodes tissues (>10 fold).^107^ ENST00000572608, ENST00000545410 (RP11-625 L16.3), and ENST00000433406 (CTC-546 K23.1) showed a significant high expression, suggesting their potential role in the pathogenesis of FL.^107^ Moreover, plenty of unknown lncRNAs, including AC00196.1, RP11-12A2.3, AF127936.5, AC010983.1, and RP11-530N7.3 has been identified in MCL, which needs their pathological role to be clear.^59^

## Concluding remarks

**Figure 6:**
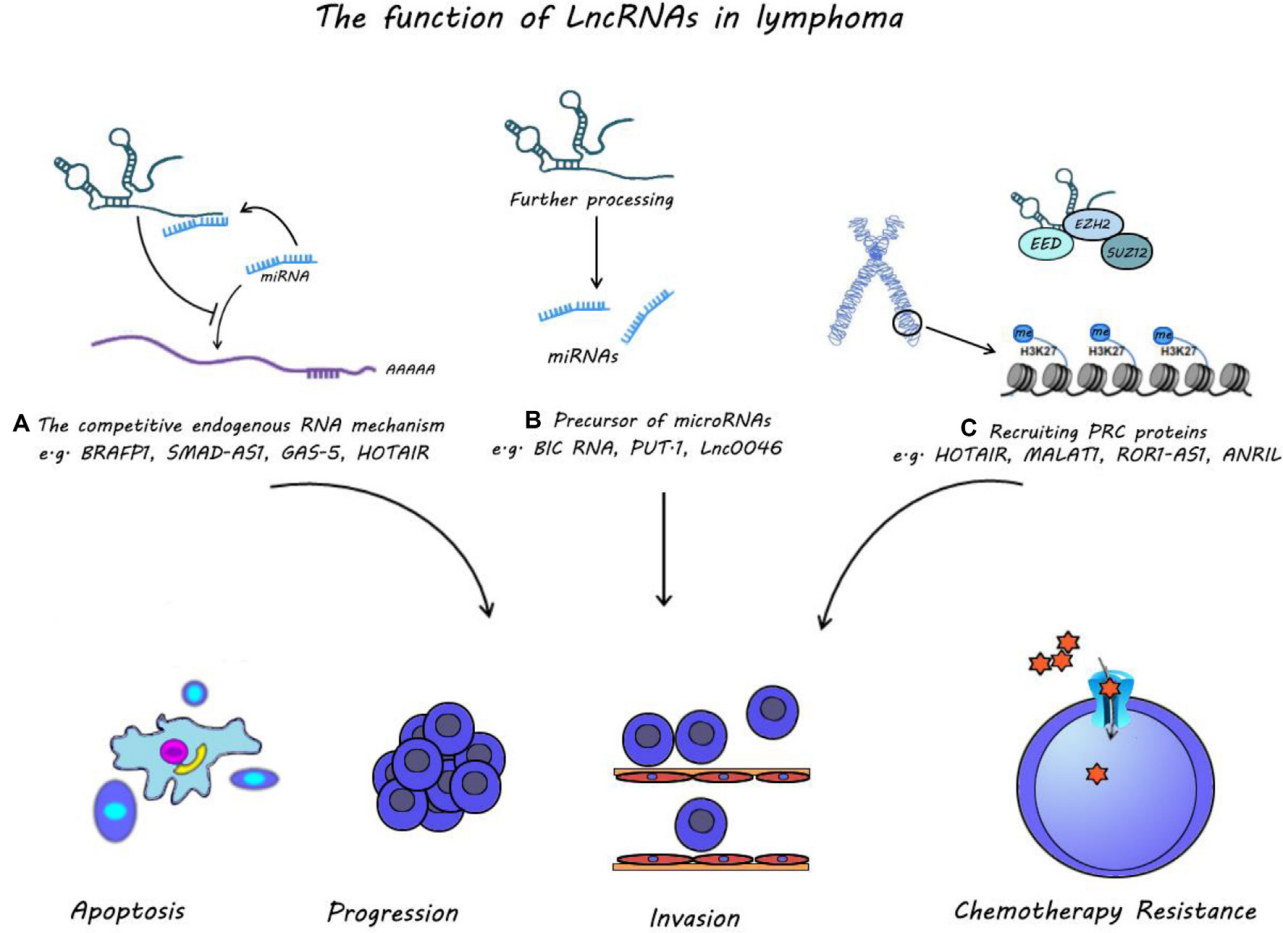
Schematic diagrams illustrating the function of lncRNAs in lymphoma cells. Various studies have determined diverse mechanisms of function by lncRNAs. **(A)** A high number of lncRNAs leads to the stabilization of mRNA by binding to miRNA. **(B)** LncRNA can act as a miRNA precursor. **(C)** LncRNAs have been known to regulate gene expression through a mechanism involving interaction with the PRC pathway. Generally, lncRNAs are imperative for lymphoma carcinogenesis by affecting apoptosis, cell proliferation, cell cycle, migration, invasion of lymphoma cells, and chemotherapy response.

Expression of LncRNAs commonly changes in lymphoma cells. LncRNAs play an important role in lymphoma carcinogenesis by affecting pathways of apoptosis, cell proliferation, and invasion (Fig. 6). Moreover, the expression level of lncRNAs can influence chemotherapy responses. These changes are related to the prognosis and survival rates of patients. Generally, lncRNA transcription is critically related to the severity and progression of lymphoma. Therefore, in the future, further in-depth research on the biological function of lncRNAs in the malignant cell may make them attractive for new therapeutic.

## Ethical approval

This article does not contain any studies with human participants or animals performed by any of the authors.

## Informed consent

For this type of study formal consent is not required.

## Conflict of interests

The authors report no conflicts of interest regarding the composition of this manuscript.
